# Neurobehavioral features in medication-overuse headache

**DOI:** 10.1016/j.ensci.2024.100538

**Published:** 2024-11-17

**Authors:** Franz Riederer, Roberto Pirrotta, Chantal Martin Soelch, Andreas R. Gantenbein, Adrian Scutelnic, Antonia Klein, Christoph J. Schankin, Peter S. Sándor

**Affiliations:** a Department of Neurology, Inselspital, Bern University Hospital, University of Bern, Switzerland; b Praxis Chreis4, Zurich, Switzerland; c Department of Psychology, University of Fribourg, Fribourg, Switzerland; d Department of Neurology, University Hospital Zurich, University of Zurich, Switzerland; e ZURZACH Care, Bad Zurzach, Switzerland

**Keywords:** Medication-overuse headache, Orbitofrontal cortex, Alexithymia, Impulsivity, Substance dependence

## Abstract

**Background:**

Medication-overuse headache (MOH) has been related to the spectrum of dependence behavior and impaired orbitofrontal cortex function. Alexithymia is a trait comprising deficits in identifying self-emotions and perception. It was the aim of the study to investigate impulsivity and alexithymia, in patients with MOH and perform correlations with cerebral grey matter.

**Material and methods:**

Patients with chronic migraine and MOH according to ICHD criteria from a tertiary headache clinic and healthy controls were investigated by a single psychiatrist, using clinical scales for self-control (BIS-11) and alexithymia (TAS-20) and screened for dependence based on DSM-IV criteria. Correlations of BIS-11 and TAS-20 with cerebral grey matter were analysed with the SPM based toolbox CAT12, using high resolution T1weighted MRI-Sequences acquired on a 3 T scanner.

**Results:**

MRI data were available from 30 MOH patients (24 women) and 47 healthy controls (26 women). MOH patients had increased impulsivity (62.2 ± 11.1 vs. 55.7 ± 7.2; *p* = 0.007) and alexithymia (49.8 ± 14.8 vs. 38.0 ± 6.5; *p* < 0.001). Analyzing only women, the results remained significant. Ninety percent of patients fulfilled DSM-IV criteria for substance dependence. There was a positive correlation between impulsivity and grey matter in the left middle orbital gyrus in healthy controls but not in patients (*p* < 0.05, corrected). No correlations with alexithymia and cerebral grey matter were found.

**Conclusions:**

The present study suggests a neurobehavioral basis for MOH, consisting of impaired impulse control, and self-perception along with features of substance dependence. Although decreased orbitofrontal cortex volume was confirmed in this MOH cohort, impulsivity and alexithymia were not correlated with this structural abnormality.

## Highlights

• Ninety percent of patients fulfilled DSM-IV criteria for substance dependence.
• Alexithymia is a trait comprising deficits in identifying self-emotions.
• Alexithymia and impulsivity are increased in MOH.
• This is not correlated with orbitofrontal cortex volume.

## Graphical abstract

**Figure:**
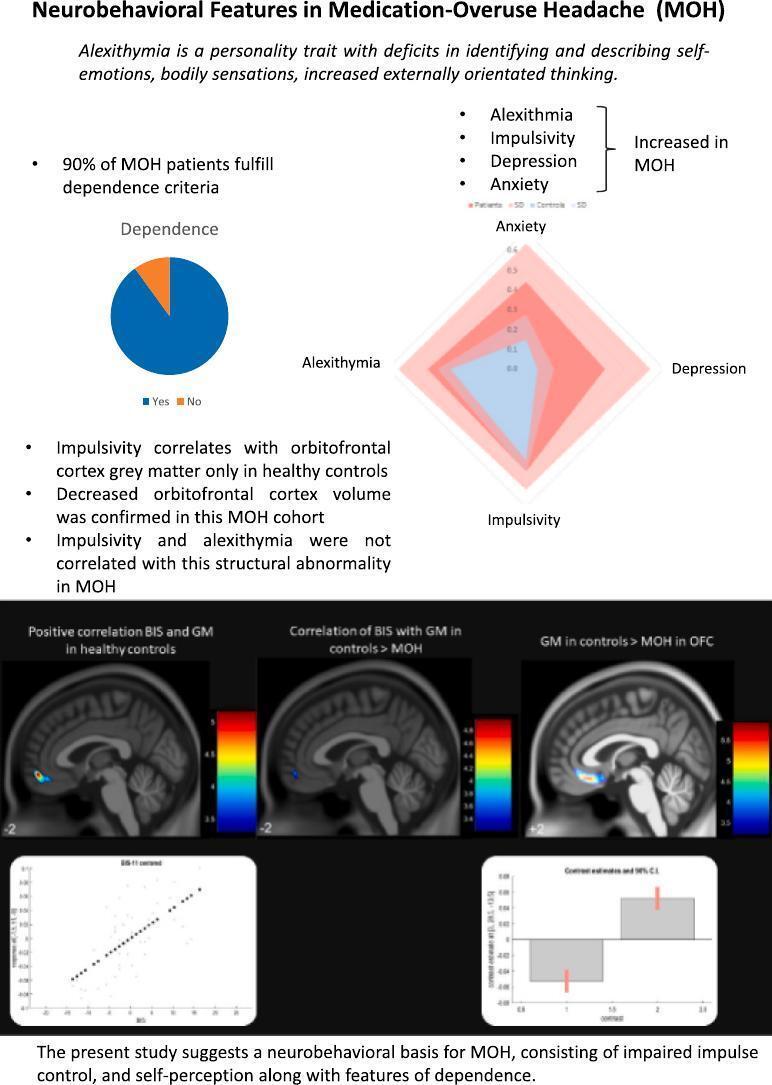

## 1 Introduction

Medication overuse headache (MOH) can develop in patients with a primary headache disorder who overuse acute medications for headache and manifests as a new headache or worsening of the primary headache [1]. The prevalence of MOH is roughly 1 % in the general population and it is associated with significant socioeconomic burden and suffering [2]. While detoxification is generally considered as important for the management of MOH, there has been an ongoing debate whether it is essential for successful treatment [3]. Botulinum toxin, topiramate, and specific migraine preventative drugs blocking CGRP are effective in chronic migraine with MOH, challenging the view that medication withdrawal is mandatory in MOH [[4], [5], [6]].

However, it has been found that detoxification as a therapeutic intervention can normalize pain thresholds which are altered in the sensitized state during medication overuse [7] and can induce neuroplastic changes in descending pain modulatory pathways [8]. Furthermore, medication-overuse headache has been related to the spectrum of dependence behavior and impaired orbitofrontal cortex function, based on psychological and neuroimaging studies [[9], [10], [11], [12]]. In present the study, we examined psychological features in MOH related to impulse control and emotional self-perception. Specifically, we investigated impulsivity and alexithymia in patients with MOH. Alexithymia is a personality trait with deficits in identifying and describing self-emotions as well as bodily sensations and increased externally orientated thinking (colloquial term “emotional blindness”) [13,14]. We performed correlations of these psychological values with cerebral grey matter, based on high-resolution-MRI. We hypothesized impaired impulse control in MOH, with relation to orbitofrontal cortex volume. Further, we expected increased alexithymia in MOH, reflecting difficulties in emotional self-perception and expression.

## 2 Material and methods

This is an extension of previously published brain morphometric studies on MOH [8,9,15]. These were focused on cross-sectional [9] and longitudinal differences [8] in cerebral grey matter, as well as correlations with anxiety and depression [9]. Patients with chronic migraine and MOH according to ICHD-II appendix diagnostic criteria [16] (the most recent criteria at the time of recruitment) and healthy controls without headache disorders were recruited from the Headache Clinic of the University Hospital Zurich, Switzerland, after approval by the ethics committee of the Canton Zurich from 2010 to 2012. Current ICHD-3 criteria [1] for MOH are almost identical to ICHD-II appendix diagnostic criteria [16]. In ICHD-3, the limit for overuse for of different drug classes without overuse of one specific class is ≥10 days per months, whereas this was ≥15 in the ICHD-II appendix criteria. Thus, included patients also fulfilled ICHD-3 criteria. Written informed consent was obtained from all study participants according to the Declaration of Helsinki. Patients were investigated by one board certified psychiatrist (R.P.) using clinical scales for self-control and impulsive personality traits (BIS-11; Barrat Impulsiveness Scale [17]) and alexithymia (TAS-20; Toronto Alexithymia Scale [13]). The TAS-20 is a self-administered questionnaire covering items for difficulties identifying and describing feelings as well as externally orientated thinking. Anxiety and depression were screened by administering the HADS-scale [18]. Dependence was investigated using the standardized face-to-face psychiatric interview, based on DSM-IV diagnostic criteria (MINI) [19].

All MRI data were acquired on a 3 Tesla Philips Achieva scanner (Philips Medical Systems) at the Institute for Biomedical Engineering, Swiss Federal Institute of Technology and University of Zurich. The T1-weighted volume sequences of the whole brain were acquired using a three-dimensional magnetization-prepared rapid acquisition gradient echo sequence (TR, 8.7 ms; TE, 2.3 ms; flip angle, 8.0°; voxel size, 0.86 × 0.86 1.0 mm, axial slice orientation, matrix size 256 × 256). Baseline data were investigated with IBM SPSS, version 28. Correlations with cerebral grey matter were analysed with the SPM (SPM12 Software - Statistical Parametric Mapping (ucl.ac.uk) based toolbox CAT12 (neuro-jena.github.io), using age, sex and total intracranial volume as covariates. Whole brain analyses were considered significant at *p* < 0.001, uncorrected, after applying an additional cluster size correction at *p* < 0.05, family-wise (FWE) error. In addition, region-of interest (ROI) analyses were performed using the orbitofrontal cortex, lateral prefrontal cortex and basal ganglia as one single ROI, at a threshold of p < 0.05 corrected for multiple comparisons with FWE. Clinical variables were compared using *t*-tests and Pearson Chi-Square tests as appropriate.

## 3 Results

**Fig. 1:**
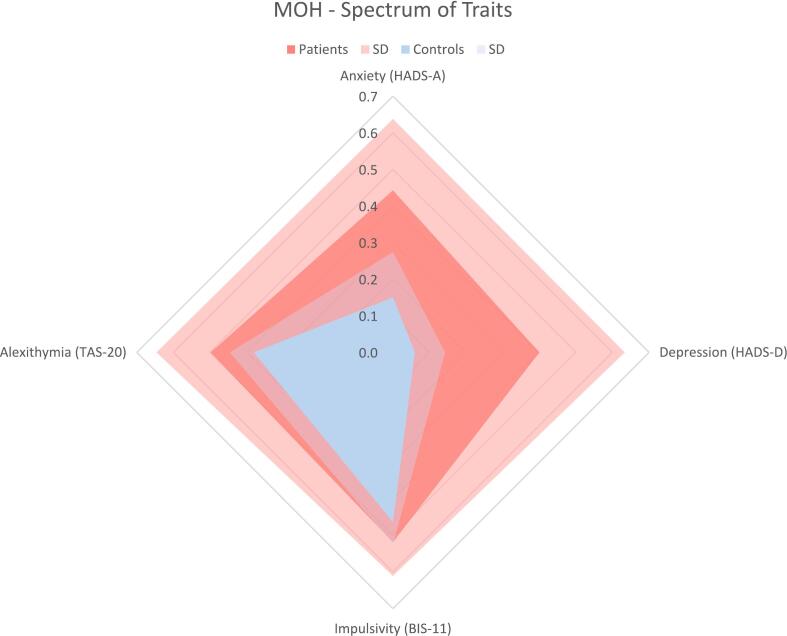
Spectrum of traits in MOH. Values and standard deviations (SD) are displayed as the relative proportions to the maxima of respective scales.

**Fig. 2:**
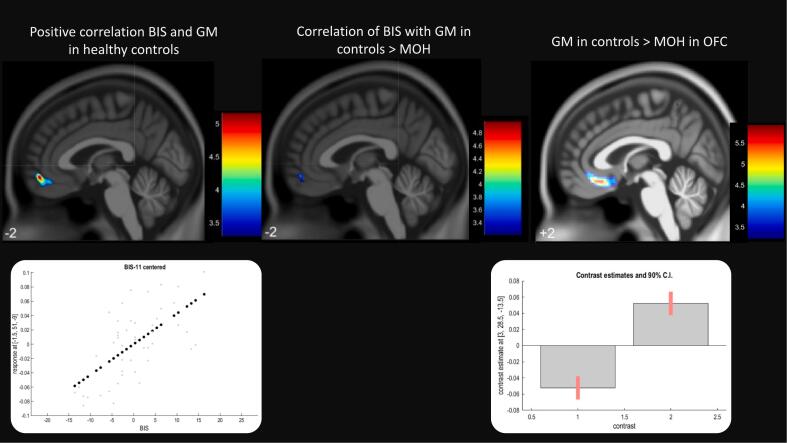
Correlation of impulsivity (BIS-11) with grey matter in the left middle orbital gyrus in healthy controls, displayed at the peak (indexed with MNI-coordinates). The color scale indicates the t-score (left image, *p* < 0.05 corrected for multiple comparisons with FWE at cluster level. No correlation was found in MOH (regression slope was significantly increased in controls (middle image, *p* < 001, uncorrected). Significantly decreased orbitofrontal cortex volume was confirmed in the present MOH cohort (p < 0.05, [FWE whole brain, displayed at *p* < 0.001 uncorrected]). Contrast estimates and 95 % CI are displayed below the image.

Thirty patients with MOH and 47 healthy controls were included. There were no significant age differences between patients and controls (Table 1). In the patient group, there were significantly more women (Table 1). MOH patients had increased impulsivity, alexithymia, anxiety and depression (Table 1, Fig. 1). Analyzing only women, the difference in psychometric variables remained significant (Table 1). Ninety percent of patients fulfilled the DSM-IV diagnostic criteria for substance dependence. There was a positive correlation between impulsivity and grey matter in the left middle orbital gyrus in healthy controls but not in patients (*p* < 0.05, corrected, see Table 2). Modelling the interaction, correlation with BIS-11 was greater in controls (*p* < 0.001, uncorrected) in the same region (Fig. 2). No correlations with alexithymia were found. Post hoc analyses confirmed reduced orbitofrontal cortex volume in MOH (Fig. 2, peak MNI coordinates x = 3, y = 28, −14, p < 0.05 FWE corrected).

**Table 1:** Demography and clinical variables in MOH patients and healthy controls. F: female.

	MOH, *N* = 30	Controls, *N* = 47	*p*-value
Age	41.6 ± 10	40.2 ± 12.6	0.590 F: 0.535
Female	24	26	0.027
TAS-20	49.8 ± 14.8	38.0 ± 6.5	<0.001 *F: 0.002*
BIS-11	62.2 ± 11.1	55.7 ± 7.2	0.007 *F: 0.036*
HADS-A	9.3 ± 4.08	3.2 ± 2.6	<0.001 *F: <0.001*
HADS-D	8.4 ± 4.94	1.3 ± 1.7	<0.001 *F: <0.001*
Dependence	18/20 (90 %)	0/47	<0.001
Nicotine	6/21 (29 %)	0 (*N* = 44)	<0.001
Headache days/months	25.7 ± 5.6 (*N* = 27)		
Triptans	10/13 (77 %)		
NSAIDs	6/13 (46 %)		
Combination analgesics	2/13 (15 %)		
Opioids	1/21 (0.05 %)		
Antiepileptic medication	10/26 (38 %)		
Antidepressants	3/20 (0.15 %)

**Table 2:** Correlation of psychometric variables with cerebral grey matter. K = cluster size, T = T-Score.

	Correlations		MNI Coordinates	k	T	Whole brain	ROI
	MOH	Controls					
Alexithymia	none	none					
BIS	none	positive	−1.5 51–9 Left middle orbital gyrus	374	5.16	*p* = 0.045	*p* = 0.029

## 4 Discussion

In the investigated prospective cohort with MOH, we found increased impulsivity and alexithymia compared to healthy controls. Impulsivity correlated with cerebral grey matter in the orbitofrontal cortex only in healthy controls, and not in MOH patients. Consistently, the regression slope was increased in controls as compared to the MOH cohort, indicating a greater correlation in controls. We found no correlations between alexithymia and cerebral grey matter.

Impulsivity has been proposed as a psychological trait in MOH in the context of substance dependence with deficits in self-control. Previous studies on impulsivity in MOH were somewhat heterogeneous [20] [21] [22], depending on study cohorts and methods. In one study, increased impulsivity was seen in a subscale of the Difficulties on Emotion Regulation Scale, but not in the BIS-11 [22]. Radat found increased impulsivity in the Iowa Gambling Task only in the subgroup of patients who were overusing opioids [20]. The positive correlation of cerebral grey matter with impulsivity in healthy controls is consistent with a previous study showing a positive correlation with limbic regions such as medial orbitofrontal and medial prefrontal cortex including the anterior cingulate cortex [23]. However, further studies on the relation between impulsivity and grey matter yielded mixed results [24,25], the majority of studies showing negative correlations between prefrontal cortical regions and trait impulsivity. Methodological issues, such as the nuisance variables used or the statistical thresholds applied for analyses can explain inconsistencies between studies. The lack of correlation between trait impulsivity and grey matter in our MOH cohort was unexpected. However, distinct association patterns between impulsivity and cerebral grey matter in healthy controls and clinical samples have been described previously [24]. It could be speculated that the lack of correlation is related to dysfunctional orbitofrontal cortex. Previous studies showed decreased grey matter and persistent hypometabolism in in orbitofrontal cortex [9,12,26] in MOH, interpreted as orbitofrontal hypofunction. In our study cohort, post hoc analyses confirmed reduced orbitofrontal cortex volume in MOH.

Consistent with our findings, alexithymia seems to be a consistent psychological trait in MOH based on chronic migraine [27,28] [29]. Alexithymia is a personality trait with deficits in identifying and describing self-emotions that has been related to dependence disorder previously [13,14,30]. It is not related to a specific psychiatric disorder [14]. In our data, there was no correlation with cerebral grey matter, including orbitofrontal regions. Our findings indicate that alexithymia and impulsivity belong to the phenotypical spectrum of MOH, comprising also anxiety, depression, substance dependence, catastrophizing, and traumatic life events [9,10,20,29,31,32]. The majority of our MOH patients fulfilled DSM-IV criteria for substance dependence, in line with previous studies [10,11,20,31]. Psychological variables such as anxiety or pain catastrophizing correlated with grey matter changes in pain modulating regions in previous studies [9,32]. We suggest that impaired impulse control and alexithymia are important predisposing or disease perpetuating factors in MOH. Impaired impulse control may interfere with unfavorable decision making towards short term goals (e.g. immediate relief from moderate headache pain) in the context of dependence [33], whereas alexithymia may interfere with the perception of bodily needs, interfering with regenerative strategies [27]. This complex phenotype in many MOH patients necessitates careful evaluation to guide a multimodal treatment approach. In line with this, clinical trials have shown beneficial effects of behavioral intervention, mindfulness or multidisciplinary programs in addition to acute medication withdrawal [[34], [35], [36]].

A limitation of the study is the lack of exact sex matching, as paticipant recruitment for psychiatric evaluation had to be accomplished within certain time constraints. However, as sex and total intracranial volume were included as covariates for voxel-based analyses, this most likely does not impact our results. Differences between psychometric variables remained significant when analyzing only women, who represented the majority of our patients. Further, psychological variables such as alexithymia were found increased in other chronic pain syndromes such as for instance in fibromyalgia [37]. Finally, it has to be kept in mind that psychiatric comorbidities in chronic migraine with MOH can improve significantly upon treatment, consistent with a bidirectional relationship. Only longitudinal studies could further clarify this issue. However, alexithymia seems to be a stable trait over time [30]. A strength of the study is the standardized evaluation of individual patients by a single psychiatrist, increasing internal consistency of the studied data set.

## 5 Conclusions

The present study suggests a neurobehavioral basis for MOH, consisting of impaired impulse control, and self-perception along with features of dependence. Although decreased orbitofrontal cortex volume was confirmed in this MOH cohort, impulsivity and alexithymia were not correlated with this structural abnormality. Findings of this study underpin the necessity of an individualized multimodal approach in the care of MOH patients.

## Ethics approval and consent to participate

- Approval by the ethics committee of the Canton Zurich was obtained (Protocol number E-37/2007)
- Informed consent was obtained from all study participants

## Consent for publication

Not applicable

## Funding

This study was funded by the Werner Alfred Selo Stiftung to Franz Riederer and the Swiss National Foundation to Peter S. Sándor, project no. 3200030_127606/1

## Authors' contributions

- FR recruited patients, analysed the data and wrote the first draft of the manuscript
- PR recruited patients, performed psychaitric evaluations and revised the manuscript draft
- CMS and PSS were responsible for the study concept and supervised the study.
- ARG recruited patients and critically revised the manuscript.
- AS and AK completed literature research and critically revised revised the manuscript draft.
- CJS: Critically revised revised the manuscript draft.

## CRediT authorship contribution statement

**Franz Riederer:** Formal analysis, Funding acquisition, Investigation, Methodology, Project administration, Writing – original draft. **Roberto Pirrotta:** Data curation, Investigation, Writing – review & editing. **Chantal Martin Soelch:** Conceptualization, Formal analysis, Supervision. **Andreas R. Gantenbein:** Data curation, Validation, Writing – review & editing. **Adrian Scutelnic:** Formal analysis, Writing – review & editing. **Antonia Klein:** Validation, Writing – review & editing. **Christoph J. Schankin:** Validation, Writing – review & editing. **Peter S. Sándor:** Conceptualization, Funding acquisition, Methodology, Project administration, Supervision, Validation, Writing – review & editing.

## Declaration of competing interest

None of the authors has conflicts of interest with regard to this work

## Data Availability

Data could be made available by the corresponding authors upon reasonable request, pending permission by the ethics committee of the canton Zurich
